# Two young women with severe Danon disease initially suspected to have cardiac amyloidosis: a case report and literature review

**DOI:** 10.3389/fcvm.2026.1934783

**Published:** 2026-10-26

**Authors:** Tianping Yu, Xin Xie, Xiuyi Pan, Ting Yu, Yanyan Su, Yang Li, Yucheng Chen, Xueqin Chen

**Affiliations:** 1 Department of Pathology, West China Hospital, Sichuan University, Chengdu, China; 2 Department of Pathology, West China Xiamen Hospital, Xiamen, China; 3 Department of Cardiology, West China Hospital, Sichuan University, Chengdu, China

**Keywords:** cardiomyopathy, Danon disease, endomyocardial biopsy, genetic diagnosis, LAMP2

## Abstract

**Background:**

Danon disease is a rare X-linked dominant lysosomal storage disorder caused by *LAMP2* mutations and characterized by cardiomyopathy, skeletal myopathy, and intellectual disability. This study reports two Chinese cases of Danon disease that were initially suspected as cardiac amyloidosis.

**Methods:**

Clinical data were collected, *LAMP2* genetic analysis was performed and literatures were reviewed.

**Results:**

Both patients presented with mainly cardiac symptoms in adulthood. Laboratory tests showed elevated levels of creatine kinase isoenzyme, troponin T, lactate dehydrogenase, hydroxybutyrate dehydrogenase, aspartate aminotransferase, and N-terminal pro-B-type natriuretic peptide. Cardiac MRI revealed diffuse left ventricular wall thickening and myocardial late gadolinium enhancement. Histopathology showed hypertrophied cardiomyocytes with disarray, vacuolar degeneration, enlarged, hyperchromatic nuclei, and fibrosis. Electron microscopy demonstrated autophagic vacuoles with sarcolemmal features-like structures, myelin-like figures resembling zebra bodies and accumulation of glycogen granules. Immunohistochemistry showed partial loss of LAMP2 expression. Genetic testing identified two rare heterozygous *LAMP2* variants: c.3G > T in exon 1 (variant of uncertain significance) and c.446del in exon 4 (likely pathogenic). Both patients underwent heart transplantation and remained alive at follow-up.

**Conclusions:**

These rare *LAMP2* variants expand the mutational spectrum of Danon disease and highlight the need for genetic counseling. Endomyocardial biopsy is valuable for diagnosis, while genetic testing is required for definitive confirmation.

## Introduction

Danon disease (DD) is an X-linked dominant disorder resulting from mutations in the lysosome-associated membrane protein 2 (LAMP2) gene. The classic clinical triad includes cardiomyopathy, skeletal myopathy, and intellectual disability (1). However, the presentation is often heterogeneous and can be mistaken for other forms of cardiomyopathy (2).

Endomyocardial biopsy (EMB) is primarily employed for monitoring graft rejection following heart transplantation and for the diagnostic assessment of cardiomyopathies of unknown etiology (3). Histopathological evaluation via EMB can provide supportive evidence in cases of unexplained hypertrophic cardiomyopathy (4). Thus, EMB remains a helpful procedure for elucidating pathological features and underlying causes.

This report describes two cases of Danon disease that presented as hypertrophic cardiomyopathy. Both cases were initially suspected of having cardiac amyloidosis at an outside hospital, but were subsequently diagnosed with Danon disease by integrated clinicopathological and genetic analysis.

## Materials and methods

### Clinical data collection

Detailed family history, motor milestones and past medical history of two cases were collected. The patients were evaluated via physical examination, echocardiography, electrocardiography, and cardiac magnetic resonance imaging (MRI). Serum creatine kinase levels were also measured.

### Histopathological analysis, immunohistochemistry staining, and electron microscopic study

Biopsy specimens were obtained from the patients' right ventricles before allogeneic cardiac transplantation. Sections of 5 μm thickness were used for conventional hematoxylin-eosin (H&E) staining and immunohistochemical studies. The explanted hearts were weighed and photographed. Myocardial samples were obtained from multiple sites in both ventricles and were either processed for routine histology or electron microscopy observation. Masson and Periodic acid Schiff (PAS) staining were respectively performed to show fibrosis and glycogen deposition. Congo Red staining was used to detect amyloid deposition. For immunohistochemistry, a primary antibody against LAMP2 (1:100 dilution) was obtained from Abclonal (China, A14017). A small cardiac muscle sample (2.5% glutaraldehyde fixation) of two patients was also sent for electron microscopic study (Electron Microscope model: Hitachi H7650).

### Molecular analysis and variant interpretation

We obtained 5 mL of peripheral blood and extracted genomic DNA via QIAamp DNA Mini Kit (Qiagen, Germany) and quantified with the Nanodrop 2000 (Life Technology, USA). Polymerase chain reaction (PCR) primers for exons 1–8,9a and 9b of the LAMP gene, flanking the intron-exon junctions were designed using Primer 3 software. Standard protocols were used for PCR. The experimental products were purified and then sequenced using the ABI 3730XL automatic sequencing machine (Applied Biosystems, Foster City, CA, USA).

Sequence variants were described according to Human Genome Variation Society (HGVS) recommendations using the LAMP2 MANE Select transcript NM_002294.3 (ENST00000200639.9). Genomic coordinates were reported relative to the GRCh37/hg19 reference genome. Population frequency and previously reported clinical interpretations were assessed using the Genome Aggregation Database (gnomAD v2.1.1, GRCh37/hg19), ClinVar, the Human Gene Mutation Database (HGMD Professional 2026.4), and the Leiden Open Variation Database (LOVD v3.0), all accessed on September 14, 2026. Variants were classified according to the ACMG/AMP framework incorporating recommendations from the ClinGen Sequence Variant Interpretation Working Group.

### Literature search for previously reported cases of danon disease in Chinese

We performed a systematic literature search in PubMed, Web of Science (SCI), CNKI, VIP, and Wanfang Database using the keywords “Danon disease” or “LAMP2”. Duplicate cases of Chinese patients were excluded from both English and Chinese articles. Duplicate cases were defined as those sharing identical diagnostic hospital, age at diagnosis, sex, and location of the LAMP2 gene variant. The search was updated through August 2026.

## Results

### Clinical history

Patient 1, a 23-year-old woman, reported exertional palpitations for more than one year, accompanied by dizziness and bilateral lower-limb weakness. In October 2020, she experienced syncope after intense exercise. Upon regaining consciousness, she complained of chest tightness, palpitations, and dizziness, but denied headache, chest pain, limb convulsions, or incontinence. Local evaluation showed elevated myocardial enzymes and echocardiographic myocardial hypertrophy; metoprolol was initiated. Over the preceding months, the patient experienced recurrent episodes of symptoms, including palpitations, chest tightness, dizziness, and lower limb weakness, which were typically exacerbated by physical exertion. She sought medical attention at a local hospital on multiple occasions during this period; however, detailed information regarding the specific treatments administered or medications prescribed was not available. The patient was subsequently referred to the outpatient department of our hospital with a suspected diagnosis of cardiac amyloidosis.

On admission in October 2021, the patient appeared thin. Manual Muscle Testing showed grade 4 strength in both lower limbs and grade 5 in both upper limbs (UK Medical Research Council grading system). She had recurrent exertional syncope, normal cognition, had completed undergraduate education, and had no family history of similar symptoms.

Laboratory investigations showed abnormalities in several markers: creatine kinase (CK, 148 IU/L; normal 20–140 IU/L), creatine kinase isoenzyme (CK-MB, 6.42 ng/mL; normal <2.88 ng/mL), troponin T (TnT, 61.0 ng/L; normal 0–14 ng/L), lactate dehydrogenase (LDH, 686.0 IU/L; normal 120–250 IU/L), hydroxybutyrate dehydrogenase (646.0 IU/L; normal 72–182 IU/L), aspartate aminotransferase (AST, 85.0 IU/L; normal <35 IU/L), and N-terminal pro-B-type natriuretic peptide (NT-proBNP, 2840.0 ng/L; normal 0–153 ng/L). Immunoglobulin, serum free light chains, immunofixation electrophoresis, and urine light-chain analysis were unremarkable.

The electrocardiogram (ECG) exhibited sinus rhythm, biventricular high voltage, and ST-T changes (Supplementary materials). Cardiac MRI revealed cardiomegaly, predominantly due to left ventricular enlargement, diffusely thickened left ventricular wall (maximum end-diastolic thickness approximately 2.4 cm), mildly narrowed left ventricular outflow tract, and patchy mid-myocardial late gadolinium enhancement, most prominent in the lateral wall. Left ventricular ejection fraction (LVEF) was 57.4%, with an end-diastolic volume (EDV) of 107.7 mL, end-systolic volume (ESV) of 45.9 mL, and stroke volume (SV) of 61.8 mL. Right ventricular ejection fraction (RVEF) was 50.8%, with EDV 94.0 mL, ESV 46.3 mL, and SV 47.7 mL. After Danon disease was diagnosed based on clinicopathological and genetic testing, orthotopic heart transplantation was performed in March 2022. This decision was made because, despite medical therapy, the patient remained in NYHA functional class III and symptoms did not improve substantially. Accordingly, an informed and voluntary decision was made despite preserved LVEF. While awaiting transplantation, she received aspirin and ivabradine; in January 2022, betahistine mesylate was added for dizziness and famotidine plus mosapride citrate for nausea (Figure 1).

**Figure 1:**
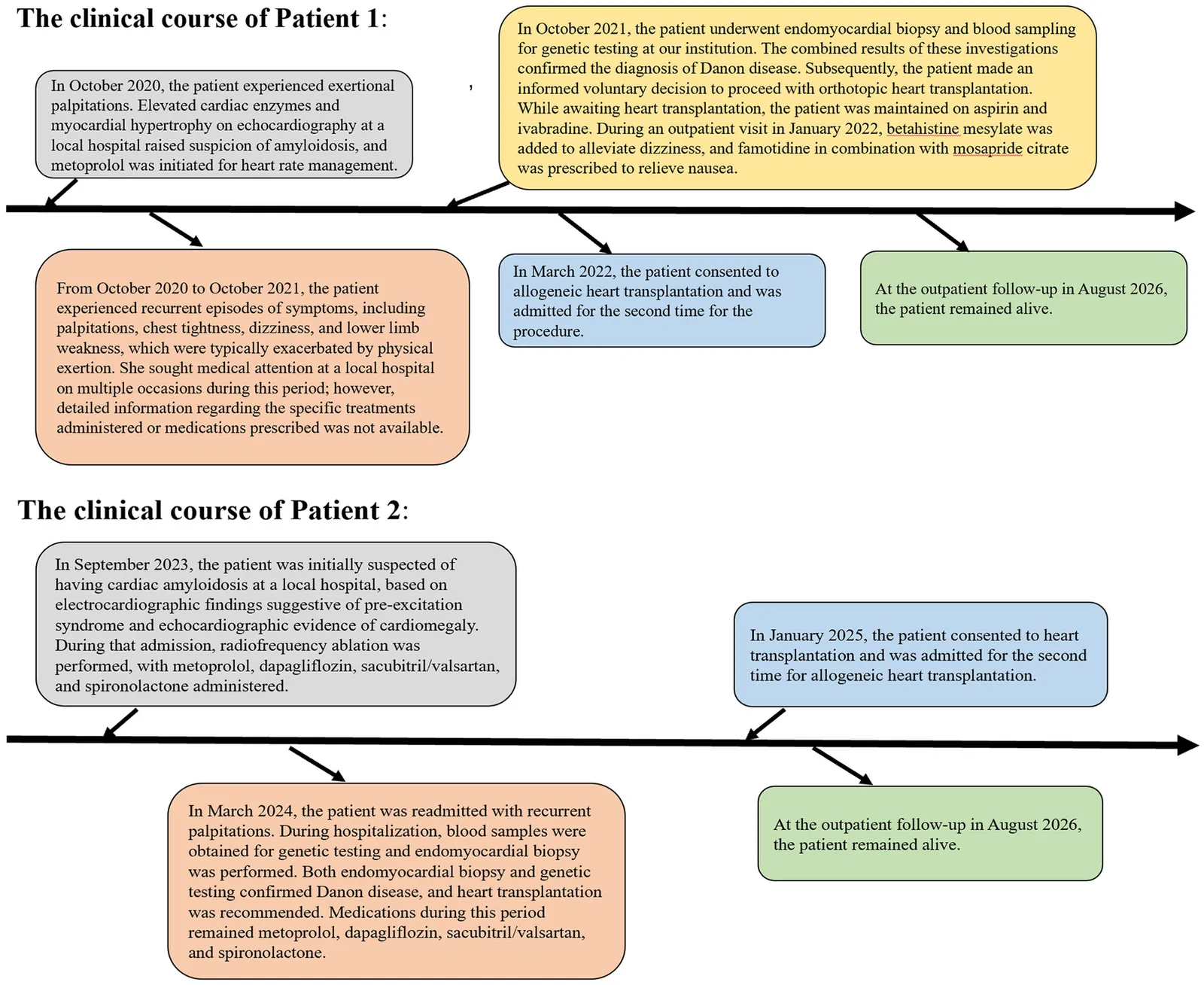
The clinical course of two patients.

Patient 2, a 21-year-old woman, presented with paroxysmal palpitations for more than one month. In September 2023, cardiac amyloidosis was suspected locally based on ECG findings suggestive of pre-excitation syndrome and echocardiographic cardiomegaly. Radiofrequency ablation was performed, and metoprolol, dapagliflozin, sacubitril/valsartan, and spironolactone were administered. Her palpitations then worsened. In March 2024, she was referred to our cardiology department. She was hemodynamically stable, cognitively normal, attending college, and had no skeletal muscle symptoms; her parents had no family history of cardiac, skeletal muscle, or mental disorders.

Laboratory tests showed CK-MB 3.49 ng/mL, TnT 50.4 ng/L, LDH 314.0 IU/L, hydroxybutyrate dehydrogenase 275.0 IU/L, AST 40.0 IU/L, and NT-proBNP 2636.0 ng/L. IgA was 3170 mg/L (836–2900), lambda light chain 6.79 g/L (3.80–6.50), and the kappa/lambda free light chain ratio was decreased at 1.40 (1.50–2.56). CK, immunofixation electrophoresis, and urine light-chain analysis were unremarkable.

ECG showed intraventricular conduction delay, frequent premature ventricular complexes, and nonspecific ST-T changes (Figure 2A). Cardiac MRI showed biventricular enlargement, predominantly left ventricular, with thickening of the interventricular septum and left ventricular free wall; multiple perfusion defects and extensive late gadolinium enhancement involved these regions, with partial subendocardial, epicardial, and transmural distribution (Figures 2B,C). LVEF was severely reduced at 19.17%, with EDV 366.33 mL, ESV 296.09 mL, and SV 70.24 mL; RVEF was 3.76%, with EDV 241.78 mL, ESV 232.69 mL, and SV 9.09 mL. In January 2025, she underwent heart transplantation after Danon disease was confirmed by EMB and genetic analysis. While awaiting transplantation, she received guideline-directed metoprolol, dapagliflozin, sacubitril/valsartan, and spironolactone (Figure 1).

**Figure 2:**
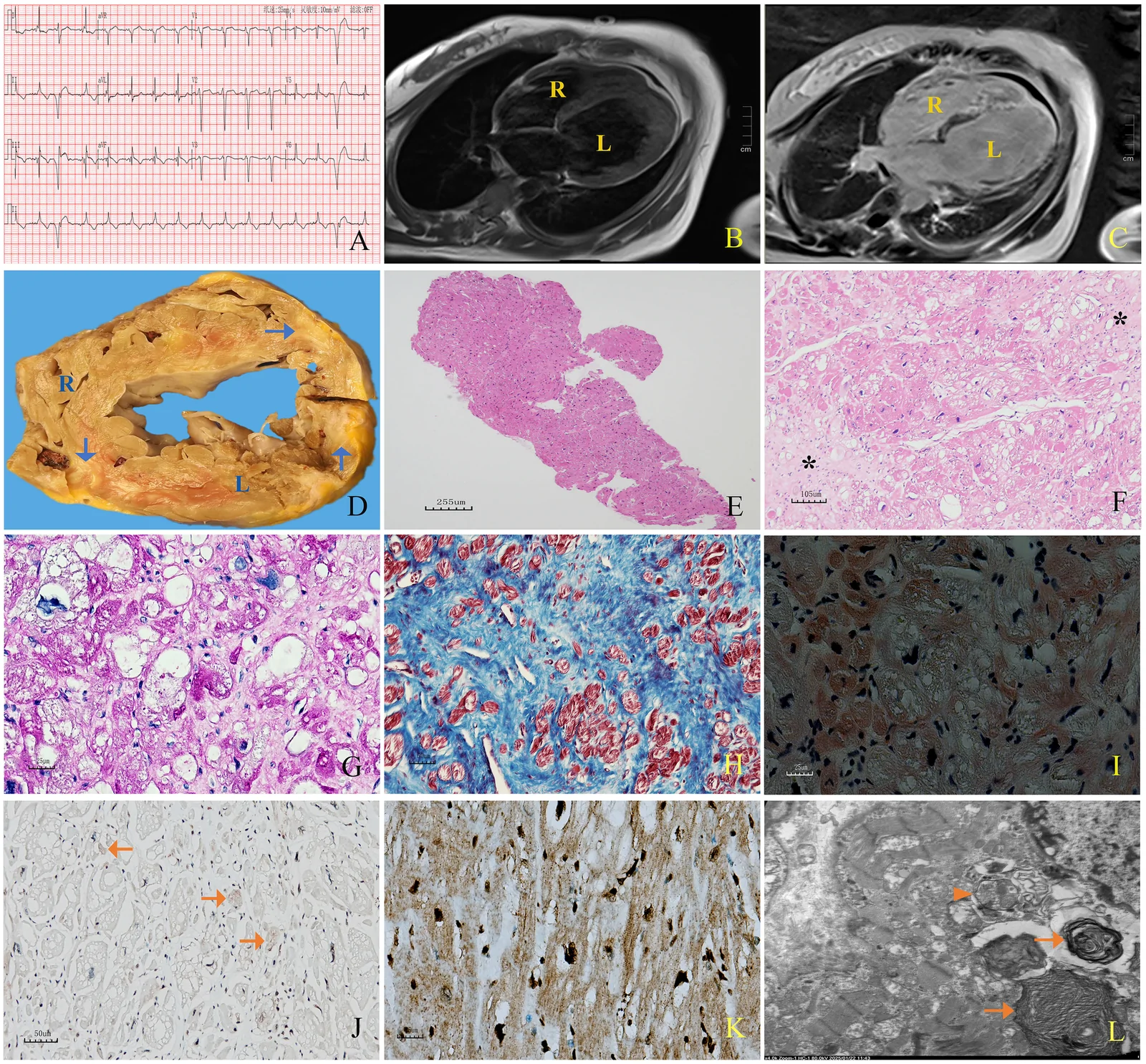
Echocardiographic, MRI, and histopathological features of the two patients. **(A)** Electrocardiogram showing sinus rhythm, intraventricular block, premature ventricular beats, and ST-T changes (Patient 2). **(B)** Cardiac MRI showing biventricular enlargement, predominantly affecting the left ventricle, with diffuse thickening of the interventricular septum and left ventricular free wall (T1WI, Patient 2). **(C)** Late gadolinium enhancement showing extensive delayed enhancement involving the interventricular septum and left ventricular free wall (Patient 2). **(D)** Short-axis section of the heart showing asymmetric hypertrophy, mainly involving the septum and left ventricular free wall (septal thickness = 2.5 cm; LV free wall thickness = 2.8 cm), with mild chamber dilation. Arrows indicate multiple small foci of myocardial scarring, mainly in the LV free wall (Patient 2). **(E)** EMB (Patient 1, HE, ×100). **(F)** Microscopic examination revealing hypertrophied cardiomyocytes with disorganized architecture and vacuolar degeneration. The asterisk (*) indicates fibrosis among cardiac myocytes (Patient 2, HE, ×100). **(G)** The vacuoles contain PAS-positive granules (Patient 1, PAS, ×400). **(H)** Masson staining showing fibrosis among cardiac myocytes (blue, Patient 1, ×400). **(I)** Congo Red staining showing no amyloid deposition (polarized light, Patient 2, ×400). **(J)** LAMP2 immunohistochemistry showing discrete clusters of unstained cardiac myocytes. Some scattered, relatively normal myocardial cells remain stained (arrows) (Patient 1, ×200). **(K)** Control hypertrophic cardiomyocytes remain stained by LAMP2 immunohistochemistry (cytoplasmic positivity, ×400). **(L)** Autophagic vacuoles with sarcolemmal features-like structures in cardiac myocytes (Patient 2, ×4000, arrowheads). Some myelin-like structures resembling zebra bodies (Patient 2, ×4000, arrows).

Both patients remained alive at 53 months and 19 months after transplantation, respectively.

### Morphological and immunohistochemical analysis of EMB and explanted heart

On gross examination, the explanted hearts weighed 450 g in Patient 1 and 440 g in Patient 2 (normal 230–280 g). Patient 1 had diffuse left ventricular wall thickening (maximum approximately 2.9 cm) and narrowing of the left ventricular outflow tract (Supplementary materials). Patient 2 had biventricular enlargement and asymmetric left ventricular hypertrophy, mainly involving the interventricular septum and free wall, with maximum thickness approximately 2.8 cm (Figure 2D). Both patients had multiple small myocardial scars involving the subendocardial and midventricular layers of the anterior, lateral, and posterior left ventricular free wall (Figure 2D). Numerous mixed thrombi were present, particularly in the right atrium and ventricle of Patient 2. Cardiac valves and coronary arteries were unremarkable.

Microscopic examination revealed hypertrophied cardiomyocytes with architectural disarray and vacuolar degeneration (Figures 2E,F), enlarged, hyperchromatic, irregular nuclei, and multifocal fibrosis (Figure 2F), with mild lymphocytic infiltration at fibrotic margins in Patient 1 (Supplementary materials). PAS staining showed glycogen accumulation within some cells (Figure 2G). Masson staining further revealed fibrosis between cardiomyocytes (Figure 2H). Congo Red staining showed no amyloid deposition (Figure 2I). Immunohistochemical analysis indicated partial loss of LAMP2 protein expression, suggestive of Danon disease (Figure 2J). Notably, LAMP2 protein exhibited cytoplasmic positivity in control hypertrophic cardiomyocytes (Figure 2K). Electron microscopy demonstrated myofibrillar disorganization, autophagic vacuoles with sarcolemmal features (AVSF)-like structures, and subsarcolemmal glycogen granules in Patient 1(Supplementary materials). AVSF-like structures and myelin-like figures resembling zebra bodies were observed in Patient 2 (Figure 2L).

### Genetic alterations and pathogenicity assessment

The LAMP2 variants identified in patient 1 and patient 2 were NC_000023.10.119603022C > A (GRCh37/hg19) (NM_002294.3.3G > T, p.Met1?) (Figure 3A) and NC_000023.10.119582935del [NM_002294.3.446del, p.(Asp149AlafsTer34)] (Figure 3B), respectively. Neither variant was detected in the proband's mother (Figure 3C).

**Figure 3:**
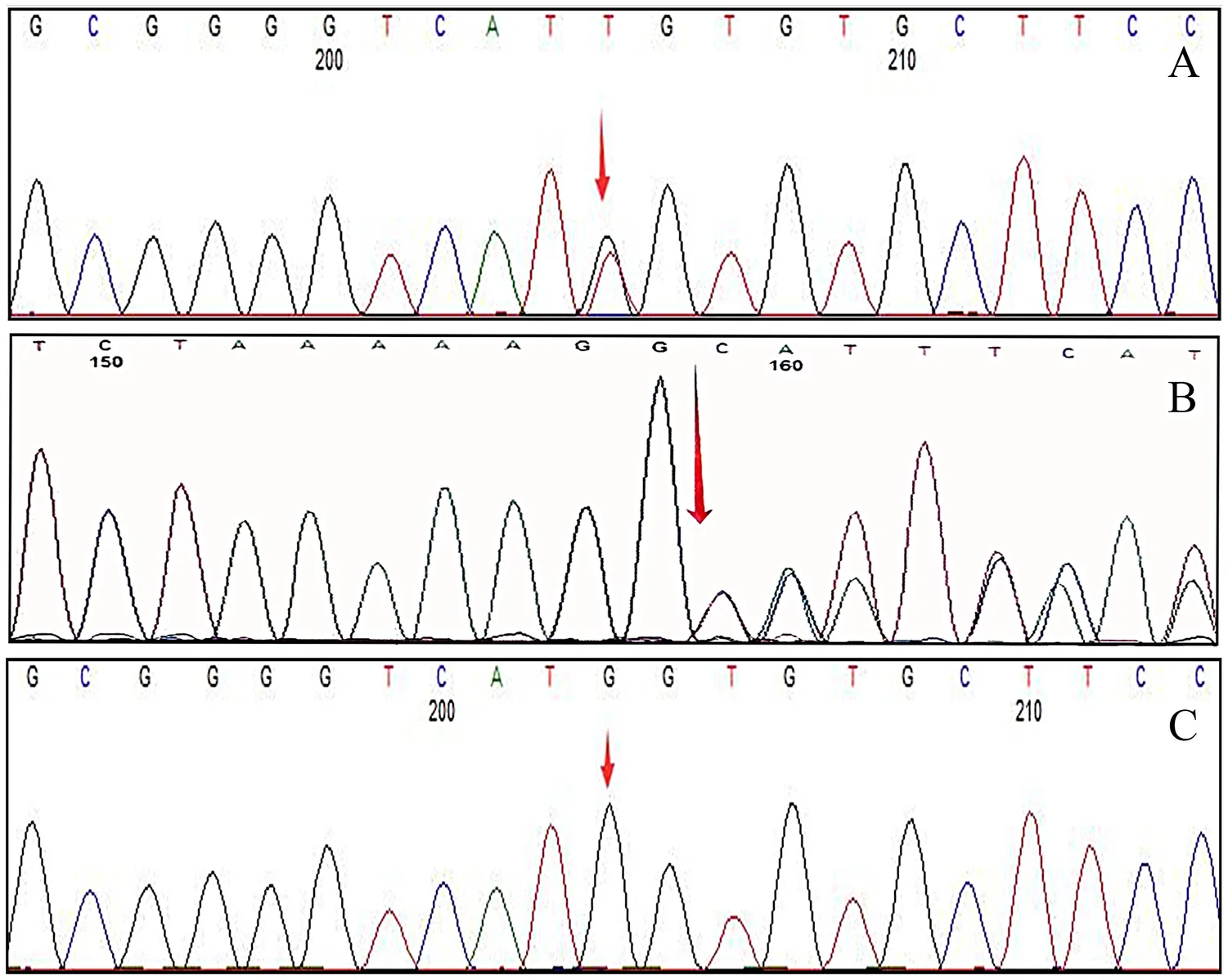
Genetic alterations of the two patients. **(A)** A heterozygous variant in exon 1 of the LAMP2 gene (c.3G > T) of Patient 1. **(B)** A heterozygous variant in exon 4 (c.446del) of Patient 2. **(C)** No variants were not detected in the proband's mother.

In patient 1, an initiation-codon variant, c.3G > T was absent from gnomAD v2.1.1, ClinVar, HGMD Professional 2026.4, and LOVD v3.0. Under the ACMG/AMP framework incorporating ClinGen SVI recommendations, PVS1_Moderate and PM2_Supporting were applied, and the variant was classified as a variant of uncertain significance. In Patient 2, a frameshift variant, c.446del was also absent from these databases; it is predicted to introduce a premature termination codon and result in nonsense-mediated mRNA decay. PVS1 and PM2_Supporting were applied, and the variant was classified as likely pathogenic. The detailed evidence codes and rationales are summarized in Table 1.

**Table 1:** ACMG/AMP interpretation of the LAMP2 variants.

Patient	Variant	Consequence	ACMG/AMP criterion	Rationale	Classification
Patient 1	NM_002294.3:c.3G > T, p.Met1?	Initiation-codon variant	PVS1_Moderate	The canonical initiation codon is disrupted. The nearest downstream in-frame Met is Met48, and pathogenic LAMP2 variants, including c.121del, p.(Cys41fs), have been reported between Met1 and Met48; PVS1 was therefore applied at Moderate strength according to ClinGen SVI guidance.	VUS
			PM2_Supporting	Absent from gnomAD v2.1.1.	
Patient 2	NM_002294.3:c.446del, p.Asp149AlafsTer34	Frameshift	PVS1 (Very Strong)	Predicted premature termination and NMD; loss of function is an established mechanism of LAMP2-related Danon disease.	Likely pathogenic
			PM2_Supporting	Absent from gnomAD v2.1.1.	

### Danon disease in Chinese patients

As of August, 2026, 115 Chinese cases had been reported (Supplementary Table). Patient ages ranged from 0.25 to 52 years, with a median onset age of 15 years and a male-to-female ratio of 4.2:1. Among the 59 cases with documented mental status, 20 patients had intellectual disability—all male. Ventricular pre-excitation was reported in 46 cases (41 males, 15 females). Follow-up data were available for 74 patients, among whom 16 had died (15 males, 1 female). The most frequent genetic variant was c.963G > A, and the most commonly affected genomic region was exon 8 (32 cases, 27.8%), followed by exon 3 (13 cases, 11.3%) and exon 6 (11 cases, 9.6%). The two variants identified in our patients—LAMP2 c.3G > T and LAMP2 c.446del, located in exon 1 and exon 4, respectively—were rare. We summarized the clinical profiles of Chinese Danon disease patients with exon 1 and exon 4 mutations in Table 2.

**Table 2:** The clinical profiles of Chinese Danon disease patients with exon 1 and exon 4 mutations.

Cases	Sex	Age(years)	Gene alterations	Locations	Consequence	Intellectual disability	VP	Follow time(months)	Died	Reference
1	M	17	c.29_35dupCGGGCTC	Exon 1	Frameshift	/	Y	/	/	Yan J, et al. (10)
2	M	17	c.29_35dupCGGGCTC	Exon 1	Frameshift	Y	Y	/	Y	Xie J, et al. (11)
3	M	24	c.35C > A	Exon 1	Nonsense	Y	/	24	Y	Xie J, et al. (11)
4	F	20	c.2T > C	Exon 1	Initiation codon variant	/	/	/	/	Wang Y, et al. (12)
5	F	2	c.64G > T	Exon 1	Nonsense	N	Y	12.0	N	Zhang Q, et al. (9)
6	M	12	Exon 1	Exon 1	Deletion	N	/	/	/	Yu K, et al. (13)
7	F	23	c.3G > T	Exon 1	Initiation codon variant	/	N	53.0	/	Yu T, et al((the present report)
8	M	15	c.467_470delTATCinsG	Exon 4	Frameshift	Y	/	1	Y	Li Y, et al. (14)
9	M	0.5	c.467 T > G	Exon 4	Nonsense	/	N	60	N	Zhang L, et al. (15)
10	M	18	c.467T > G	Exon 4	Nonsense	/		17	N	Pu L, et al. (16)
11	M	0.25	c.467T > G	Exon 4	Nonsense	N	N	14.0	N	Zhang Q, et al. (9)
12	M	4	c.507G > A	Exon 4	Nonsense	/	N	2.2	/	Zhang Q, et al. (9)
13	M	12	c.520C > T	Exon 4	Nonsense	/	Y	0.17	/	Zhang Q, et al. (9)
14	M	16	c.520C > T	Exon 4	Nonsense	/	Y	1.8	/	Zhang Q, et al. (9)
15	F	21	c.446del	Exon 4	Frameshift	/	N	19.0	/	Yu T, et al((the present report)

VP, ventricular pre-excitation.

## Discussion

Danon disease is a rare X-linked dominant genetic disorder first described by Danon et al. in 1981 (5). The initial report involved two 16-year-old boys who presented with the classic triad of intellectual disability, cardiomyopathy, and skeletal myopathy. Muscle biopsy and electron microscopy revealed intra lysosomal glycogen accumulation with normal acid *α*-glucosidase activity. The prevalence of Danon disease in the general population remains uncertain; however, it accounts for approximately 4% of childhood hypertrophic cardiomyopathy cases (6) and up to 17% of cases featuring left ventricular hypertrophy with pre-excitation syndrome (7). The age at diagnosis ranges from birth to 42 years in males and from 2 to 65 years in females (8). Males tend to present at a younger age and may experience more severe multisystem involvement compared to females (8, 9). Additionally, pediatric patients with ventricular pre-excitation (VP) often exhibit worse cardiac function and more pronounced hypertrophy than those without VP (9). These findings are consistent with the clinical characteristics observed in previously reported Chinese patients. The two variants identified in our study—LAMP2 c.3G > T and c.446del, situated in exon 1 and exon 4, respectively—are considered rare. Including our cases, only 15 such cases (9–16)—all involving young patients—have been reported in China. Our cases therefore contribute meaningful data to the ongoing investigation of Danon disease.

In the two cases we reported, both patients were female and developed symptoms only in adulthood. Clinical manifestations were mainly cardiac symptoms. Patient 2 had elevated IgA, elevated lambda light chain, and a reduced kappa/lambda free light chain ratio, requiring differentiation from cardiac amyloidosis. Therefore, it is helpful to perform an EMB in a timely manner.

Common histopathological features in EMBs or explanted hearts include myofibrillar disarray, cardiomyocyte hypertrophy, enlarged or pyknotic nuclei, cytoplasmic vacuolization, macrophage accumulation, and necrotic changes (17). Electron microscopy reveals that LAMP2 deficiency disrupts autophagy, leading to massive accumulation of undigested material within lysosomes. Myelin-like figures resembling zebra bodies are frequently observed (18). Accumulations of glycogen in cardiac and skeletal muscle, as well as lipid storage in hippocampal neurons, may also be present (19). Immunohistochemical staining for LAMP2 typically shows a mosaic pattern, with clusters of both stained and unstained cardiomyocytes, the latter being more common in the interventricular septum (20). In our two patients, we observed asymmetric left ventricular hypertrophy on gross examination; cytoplasmic vacuolization and fibrosis on light microscopy; autophagic vacuoles and myelin-like figures by electron microscopy; and absent LAMP2 staining by immunohistochemistry. These findings were suggestive of Danon disease, a diagnosis that was further confirmed by genetic testing.

The two LAMP2 variants differed in the strength of genetic evidence supporting pathogenicity. The c.3G > T variant disrupts the canonical initiation codon. Under the ClinGen SVI PVS1 framework, initiation-codon variants require caution because translation may reinitiate downstream. In NP_002285.1, the nearest downstream in-frame methionine is Met48. Because pathogenic LAMP2 variants have been reported between Met1 and Met48, including c.121del, p.Cys41fs, PVS1 was applied at Moderate rather than Very Strong. With PM2_Supporting, the evidence was insufficient for likely pathogenic classification, so c.3G > T remained a variant of uncertain significance. In contrast, c.446del causes a frameshift and introduces a premature termination codon. Given predicted nonsense-mediated mRNA decay and the established loss-of-function mechanism of LAMP2-related Danon disease, it meets PVS1 Very Strong; with PM2_Supporting, it is likely pathogenic.

Danon disease is caused by loss-of-function mutations in the LAMP2 gene. In 2000, Ichizo Nishino et al. (21) first identified through genetic and protein analyses that defects in LAMP2, particularly the LAMP2b isoform, underlie the disease. The LAMP2 gene is located at Xq24 and contains an open reading frame of 1,233 nucleotides encoding 410 amino acids. Exons 1–8 and part of exon 9 encode the luminal domain, while the remainder of exon 9 encodes the transmembrane domain and cytoplasmic tail (22). Alternative splicing of exon 9 yields three isoforms: LAMP2a, LAMP2b, and LAMP2c. LAMP2a and LAMP2c are widely expressed, with high abundance in the liver, lungs, and placenta. LAMP2a is involved in chaperone-mediated autophagy, and LAMP2c facilitates RNA and DNA degradation in the brain. LAMP2b is mainly involved macroautophagy and highly expressed in the heart, skeletal muscle, and brain, consistent with the classic clinical triad of Danon disease. Although most mutations affect all three isoforms, only mutations specifically impacting LAMP2b have been reported to date (23). Deficient LAMP2b leads to impaired macroautophagy, resulting in accumulation of autophagic vacuoles containing glycogen and cytoplasmic debris, thereby contributing to cardiomyocyte hypertrophy (24). To date, over 250 pathogenic LAMP2 variants have been identified in Danon disease; these are predominantly nonsense or splicing mutations, whereas missense mutations and large rearrangements are rare (9, 24).

Danon disease typically presents as hypertrophic cardiomyopathy and is often misdiagnosed without genetic testing. Timely EMB can aid in diagnosis. Common differential diagnoses include amyloidosis, Fabry disease, Pompe disease, and mitochondrial cardiomyopathy (18, 25). Amyloidosis shows atrophic cardiomyocytes, eosinophilic interstitial and vascular deposits, Congo red positivity, and randomly oriented 10-nm amyloid fibrils. Fabry disease, an X-linked *α*-galactosidase A disorder, shows cardiomyocyte hypertrophy and vacuolation, with concentric lamellar myelin figures of 4.5-nm periodicity. Pompe disease, or adult-onset glycogen storage disease type II, results from *α*-glucosidase deficiency and shows glycogen accumulation and vacuolar degeneration. Mitochondrial cardiomyopathy shows mitochondrial proliferation or depletion, vacuolar degeneration, glycogen accumulation, disrupted cristae, and membrane irregularities.

Limitations of this study include the following. First, genetic testing was not performed on all available relatives of the two probands, and thus the *de novo* status of the variants could not be conclusively established. Second, the pathogenicity of the identified genetic alterations was assessed only at the in silico level; no functional validation, such as RNA analysis or protein blotting, was carried out to support the predictions due to lack of available tissue. Third, this study was a retrospective observational review of published cases and may be subject to publication and referral bias.

In conclusion, Danon disease is challenging to recognize due to its rarity and nonspecific early manifestations. In patients whose initial presentation is primarily cardiac, EMB may serve as a useful diagnostic adjunct, whereas genetic testing is required for definitive confirmation of the underlying etiology.

## Data Availability

The datasets presented in this study can be found in online repositories. The names of the repository/repositories and accession number(s) can be found in the article/Supplementary Material.
