# Supplementary material for: Two young women with severe Danon disease initially suspected to have cardiac amyloidosis: a case report and literature review
Source: Front Cardiovasc Med. 2026 Oct 26;13:1934783. doi: 10.3389/fcvm.2026.1934783 (PMC13745680; doi:10.3389/fcvm.2026.1934783)
Supplement: Supplementary file 1 [file Table1.docx]

| **Supplementary Table1 Danon disease has been reported in Chinese people** | | | | | | | | | | | |
| --- | --- | --- | --- | --- | --- | --- | --- | --- | --- | --- | --- |
| **Cases** | **Sex** | **Age(year)** | **Gene alterations** | **Locations** | **Consequence** | **NYHA Class** | **Intellectual disability** | **VP** | **Follow time (months)** | **Died** | **References** |
| 1 | M | 15 | c.808dupG | exon 6 | frameshift | / | N | Y | / | / | 2012-Chen XL, et al.-Gene |
| 2 | M | 22 | c.892G>T | exon 7 | nonsense | / | N | Y | / | / | 2012-Hong D, et al.-Clinical Neuropathology |
| 3 | M | 16 | c.1204A>T | exon 9B | nonsense | / | N | N | / | / | 2012-Hong D, et al.-Clinical Neuropathology |
| 4 | M | 18 | c.1205Cdel | exon 8 | frameshift | / | N | Y | 5 | Y | 2013-He J, et al.-Chin J Cardiol |
| 5 | M | 18 | c.369_370TGdel | exon 3 | frameshift | / | N | N | 24 | N | 2013-He J, et al.-Chin J Cardiol |
| 6 | M | 17 | c.189–190TGdel | exon 3 | frameshift | / | N | N | 6 | N | 2014-He J, et al.-Herz |
| 7 | M | 24 | c.864G>A | exon 6 | splicing | / | Y | / | 6 | N | 2014-Luo S, et al.-Clinical Neuropathology |
| 8 | M | 21 | c.396delA | exon 3 | frameshift | / | / | Y | 6 | Y | 2014-Luo S, et al.-Clinical Neuropathology |
| 9 | M | 3 | c.973-974insC | exon 8 | frameshift | / | N | / | 14 | N | 2014-Ding Y, et al.-Chinese Journal of Practical Pediatrics |
| 10 | M | 20 | c.808dupG | exon 6 | frameshift | / | / | / | / | / | 2016-Lin Y, et al.-Chin J Ultrasonogr |
| 11 | M | 17 | c.29_35dupCGGGCTC | exon 1 | frameshift | / | / | Y | / | / | 2017-Yan J, et al.-Chinese Circulation Journal |
| 12 | M | 15 | c.467_470delTATCinsG | exon 4 | frameshift | / | Y | / | 1 | Y | 2018-Li Y, et al.-Chin J Neurol |
| 13 | F | 26 | c.974delTinsAA | exon 8 | frameshift | / | N | Y | 24 | Y | 2019-Guo S, et al.-Exp Ther Med |
| 14 | M | 23 | c.974delTinsAA | exon 8 | frameshift | / | N | N | 12 | N | 2019-Guo S, et al.-Exp Ther Med |
| 15 | M | 19 | c.661_667del | exon 5 | frameshift | / | / | Y | 25 | Y | 2019-Zhang Y, et al.-Mdeicine |
| 16 | M | 18 | c.741+1G>T | exon 5 | splicing | / | N | Y | / | / | 2019-Zhou N, et al.-Mol Genet Genomic Med |
| 17 | M | 52 | c.277G>A | exon 3 | missense | / | N | N | / | / | 2019-Xu J,et al.-Mol Genet Genomic Med |
| 18 | M | 23 | IVS 1‐2A > G | intron 1 | frameshift | / | Y | Y | / | / | 2019-Liu Y, et al.-Mol Genet Genomic Med |
| 19 | M | 8 | c.858del T | exon 6 | frameshift | II | N | Y | 60 | N | 2020-He J, et al.-Clinical Radiology |
| 20 | M | 14 | / | / | / | III | Y | Y | 24 | Y | 2020-He J, et al.-Clinical Radiology |
| 21 | M | 23 | c.973delG | exon 8 | frameshift | II | Y | Y | 6 | N | 2020-He J, et al.-Clinical Radiology |
| 22 | M | 15 | / | / | / | III | N | N | 120 | N | 2020-He J, et al.-Clinical Radiology |
| 23 | M | 19 | / | / | / | II | N | N | 6 | N | 2020-He J, et al.-Clinical Radiology |
| 24 | M | 8 | c.973delC | exon 8 | frameshift | II | Y | Y | 48 | N | 2020-He J, et al.-Clinical Radiology |
| 25 | M | 7 | c.928+2T>G | exon 7 | splicing | / | / | Y | / | / | 2020-Wu J, et al.-Electrocardiology and Circulation |
| 26 | M | 4 | c.928G>A | exon 7 | splicing | / | / | N | / | / | 2020-Zhao X, et al.-Journal of Clinical Cardiology(China) |
| 27 | M | 9 | c.877C>T | exon 7 | nonsense | / | N | / | 24 | N | 2020-Zhang Y, et al.-Guangdong Medical Journal |
| 28 | M | 4 | c.877C>T | exon 7 | nonsense | / | N | / | 10 | N | 2020-Zhang Y, et al.-Guangdong Medical Journal |
| 29 | M | 18 | c.1052delG | exon 8 | frameshift | / | Y | Y | 2 | Y | 2021-Xu J, et al.-Diagnostic Pathology |
| 30 | M | 11 | c.388G>A | exon 3 | point mutations | I | N | N | / | / | 2021-Wang X, et al.-Exp Ther Med |
| 31 | F | 3 | c.959A>G | exon 8 | point mutations | I | / | N | / | / | 2021-Wang X, et al.-Exp Ther Med |
| 32 | M | 12 | c.779_782del | exon 6 | frameshift | I | N | N | / | / | 2021-Wang X, et al.-Exp Ther Med |
| 33 | M | 20 | c.928G>A | exon 7 | splicing | I | N | Y | / | / | 2021-Wang X, et al.-Exp Ther Med |
| 34 | M | 0.5 | c.121delT | exon 2 | frameshift | I | / | Y | / | / | 2021-Wang X, et al.-Exp Ther Med |
| 35 | M | 1 | c.750delA | exon 6 | frameshift | I | / | N | / | / | 2021-Wang X, et al.-Exp Ther Med |
| 36 | F | 23 | c.1054delG | exon 8 | frameshift | III | / | N | / | / | 2021-Wang X, et al.-Exp Ther Med |
| 37 | M | 19 | c.741+2T>C | intron 5 | splicing | / | N | N | 24 | N | 2021-Li Z, et al.-Front Cardiovasc Med |
| 38 | M | 0.33 | c.973delG | exon 8 | frameshift | / | / | N | 2 | Y | 2021-Zhang L, et al.-Frontiers in Genetics |
| 39 | M | 0.5 | c.467T > G | exon 4 | nonsense | / | / | N | 60 | N | 2021-Zhang L, et al.-Frontiers in Genetics |
| 40 | M | 16 | c.963G > A | exon 8 | / | II | Y | Y | 24 | N | 2022-Wang J, et al.-Front Cardiovasc Med |
| 41 | F | 38 | c.963G > A | exon 8 | / | III | N | / | 84 | N | 2022-Wang J, et al.-Front Cardiovasc Med |
| 42 | F | 36 | c.963G > A | exon 8 | / | II | N | / | / | / | 2022-Wang J, et al.-Front Cardiovasc Med |
| 43 | M | 8 | c.963G > A | exon 8 | / | / | / | / | / | / | 2022-Wang J, et al.-Front Cardiovasc Med |
| 44 | M | 2 | c.963G > A | exon 8 | / | / | / | / | / | / | 2022-Wang J, et al.-Front Cardiovasc Med |
| 45 | F | 25 | c.764_765insGA | exon 6 | frameshift | / | N | / | / | N | 2022-Wang J, et al.-Front Cardiovasc Med |
| 46 | M | 19 | c.257_258delCC | exon 3 | frameshift | / | / | / | / | Y | 2022-Xie J, et al.-Front Cardiovasc Med |
| 47 | M | 19 | c.719_721dupAGC | exon 5 | in-frame | / | Y | Y | 24 | Y | 2022-Xie J, et al.-Front Cardiovasc Med |
| 48 | M | 17 | c.29_35dupCGGGCTC | exon 1 | frameshift | / | Y | Y | / | Y | 2022-Xie J, et al.-Front Cardiovasc Med |
| 49 | M | 15 | c.1057C>T | exon 8 | / | / | / | / | / | Y | 2022-Xie J, et al.-Front Cardiovasc Med |
| 50 | M | 24 | c.35C>A | exon 1 | nonsense | / | Y | / | 24 | Y | 2022-Xie J, et al.-Front Cardiovasc Med |
| 51 | M | 19 | c.973dupC | exon 8 | frameshift | / | Y | Y | / | Y | 2022-Xie J, et al.-Front Cardiovasc Med |
| 52 | M | 21 | c.973delC | exon 8 | frameshift | / | N | Y | 24 | Y | 2022-Xie J, et al.-Front Cardiovasc Med |
| 53 | F | 44 | c.64+1G>A | intron 1 | splicing | / | / | / | / | N | 2022-Xie J, et al.-Front Cardiovasc Med |
| 54 | M | 16 | c.963G > A | exon 8 | / | / | / | / | / | N | 2022-Xie J, et al.-Front Cardiovasc Med |
| 55 | M | 15 | c.257_258delCC | exon 3 | frameshift | / | / | / | / | Y | 2022-Xie J, et al.-Front Cardiovasc Med |
| 56 | M | 23 | c.65-2A>G | exon 2 | splicing | / | / | / | / | N | 2022-Xie J, et al.-Front Cardiovasc Med |
| 57 | M | 14 | c.808_809 insG | exon 6 | frameshift | / | / | / | / | Y | 2022-Xie J, et al.-Front Cardiovasc Med |
| 58 | M | 20 | c.64+1G>A | intron 1 | splicing | / | / | Y | / | N | 2022-Xie J, et al.-Front Cardiovasc Med |
| 59 | M | 17 | c.877C>T | exon 7 | nonsense | / | / | / | / | N | 2022-Xie J, et al.-Front Cardiovasc Med |
| 60 | M | 30 | c.883dupT | exon 7 | frameshift | / | Y | Y | 12 | N | 2022-Zhang S, et al.-Shanghai Med J |
| 61 | M | 14 | c.864+5G>A | intron 6 | splicing | / | N | Y | 24 | N | 2023-Fu D, et al.-ESC Heart Faillure |
| 62 | M | 19 | c.190_191delGT | exon 3 | frameshift | / | / | / | 60 | N | 2023-Chen F, et al.-Radiology |
| 63 | M | 10 | c.973_974insC | exon 8 | frameshift | / | / | / | 12 | N | 2023-Tang K, et al.-Cardiology in the Young |
| 64 | F | 20 | c.2T>C | exon 1 | missense | / | / | / | / | / | 2023-Wang Y, et al.-Mol Genet Genomic Med |
| 65 | M | 12 | c.973dupC | exon 8 | frameshift | / | / | / | 8 | N | 2023-Wu D, et al.-European Heart Journal |
| 66 | M | 18 | c.467T>G | exon 4 | nonsense | / | / | / | 17 | N | 2023-Pu L, et al.-J Clin Hepatol |
| 67 | M | 9 | c.973dupC | exon 8 | frameshift | / | Y | / | / | / | 2023-Zhang H, et al.-J Chin Pract Diagn Ther |
| 68 | M | 16 | c.740dupA | exon 5 | frameshift | / | Y | Y | 14 | N | 2023-Yang L, et al.-Chin J Cardiovasc Med |
| 69 | F | 37 | c.1075C > T | exon 8 | nonsense | / | / | N | 36 | N | 2024-Li X, et al.-Image focus |
| 70 | F | 51 | c.696T>A | exon 5 | / | / | / | Y | / | N | 2024-Wang N, et al.-Front Cardiovadc Med |
| 71 | F | 25 | c.733C > T | exon 5 | / | / | / | Y | / | / | 2024-Yue X, et al.-European Heart Journal |
| 72 | M | 16 | c.928G＞C |  | splicing | / | / | Y | 84 | N | 2024-Zhou Y, et al.-J Clin Electrocardiol |
| 73 | M | 18 | c.1078_1081delTCTC | exon 8 | frameshift | III | Y | Y | 1 | Y | 2024-Deng Y, et al.-MOLECULAR CARDIOLOGY OF CHINA |
| 74 | M | 11 | c.821_822delTT | exon 2 | frameshift | I | N | Y | / |  | 2024-Deng Y, et al.-MOLECULAR CARDIOLOGY OF CHINA |
| 75 | M | 14 | c.257_258delCC | exon 3 | frameshift | I | Y | Y | 108 | Y | 2024-Deng Y, et al.-MOLECULAR CARDIOLOGY OF CHINA |
| 76 | M | 16 | c.320_321 insCATC | exon 3 | frameshift | I | Y | Y | 36 | Y | 2024-Deng Y, et al.-MOLECULAR CARDIOLOGY OF CHINA |
| 77 | M | 15 | c.808_809 insG | exon 6 | frameshift | I | N | Y | / | / | 2024-Deng Y, et al.-MOLECULAR CARDIOLOGY OF CHINA |
| 78 | M | 27 | c.973delG | exon 8 | frameshift | III | N | Y | 12 | N | 2024-Deng Y, et al.-MOLECULAR CARDIOLOGY OF CHINA |
| 79 | M | 3 | c.966_978delTGCCCCCCTGGGA | exon 8 | frameshift | II | Y | N | 12 | N | 2024-Deng Y, et al.-MOLECULAR CARDIOLOGY OF CHINA |
| 80 | M | 13 | / | / | / | I | Y | Y | 84 | Y | 2024-Deng Y, et al.-MOLECULAR CARDIOLOGY OF CHINA |
| 81 | F | 21 | c.138G＞A | exon 2 | nonsense | / | N | N | / | / | 2024-Ouyang C, et al.-Chinese Journal of Arteriosclerosis |
| 82 | F | 2 | c.64G>T | exon 1 | nonsense | / | N | Y | 12.0 | N | 2025-Zhang Q, et al.-Can J Cardiol |
| 83 | M | 12 | c.64+1G>A | intron 1 | splicing | / | / | N | 14.7 | / | 2025-Zhang Q, et al.-Can J Cardiol |
| 84 | M | 4 | c.190_191delGT | exon 3 | frameshift | / | / | N | 4.3 | / | 2025-Zhang Q, et al.-Can J Cardiol |
| 85 | M | 3 | c.340_342delins20 | exon 3 | frameshift | / | / | Y | 1.7 | / | 2025-Zhang Q, et al.-Can J Cardiol |
| 86 | F | 11 | c.371_375delCAACA | exon 3 | frameshift | / | / | N | 2.1 | / | 2025-Zhang Q, et al.-Can J Cardiol |
| 87 | M | 0.25 | c.467T>G | exon 4 | nonsense | / | N | N | 14.0 | N | 2025-Zhang Q, et al.-Can J Cardiol |
| 88 | M | 4 | c.507G>A | exon 4 | nonsense | / | / | N | 2.2 | / | 2025-Zhang Q, et al.-Can J Cardiol |
| 89 | M | 12 | c.520C>T | exon 4 | nonsense | / | / | Y | 0.17 | / | 2025-Zhang Q, et al.-Can J Cardiol |
| 90 | M | 16 | c.520C>T | exon 4 | nonsense | / | / | Y | 1.8 | / | 2025-Zhang Q, et al.-Can J Cardiol |
| 91 | F | 11 | c.624delT | exon 5 | frameshift | / | / | N | 2.3 | / | 2025-Zhang Q, et al.-Can J Cardiol |
| 92 | M | 4 | c.714dup | exon 5 | frameshift | / | / | N | 0.4 | / | 2025-Zhang Q, et al.-Can J Cardiol |
| 93 | M | 13 | c.718C>T | exon 5 | nonsense | / | N | Y | 6.0 | Y | 2025-Zhang Q, et al.-Can J Cardiol |
| 94 | F | 2 | c.718C>T | exon 5 | nonsense | / | N | N | 6.0 | N | 2025-Zhang Q, et al.-Can J Cardiol |
| 95 | F | 10 | c.749C>G | exon 6 | nonsense | / | N | N | 6.0 | N | 2025-Zhang Q, et al.-Can J Cardiol |
| 96 | M | 6 | c.843T>A | exon 6 | nonsense | / | / | N | 6.4 | / | 2025-Zhang Q, et al.-Can J Cardiol |
| 97 | M | 3 | c.864+3_864+6del | intron 6 | splicing | / | / | N | 0.8 | / | 2025-Zhang Q, et al.-Can J Cardiol |
| 98 | M | 3 | c.865-2A>G | intron 6 | splicing | / | / | N | 1.4 | / | 2025-Zhang Q, et al.-Can J Cardiol |
| 99 | M | 2 | c.865-2A>G | intron 6 | splicing | / | / | N | 7.0 | / | 2025-Zhang Q, et al.-Can J Cardiol |
| 100 | F | 10 | c.865-2A>G | intron 6 | splicing | / | / | Y | 0.25 | / | 2025-Zhang Q, et al.-Can J Cardiol |
| 101 | F | 18 | c.877C>T | exon 7 | nonsense | / | / | N | 1.4 | / | 2025-Zhang Q, et al.-Can J Cardiol |
| 102 | M | 12 | c.928G>A | exon 7 | splicing | / | / | N | 2.7 | / | 2025-Zhang Q, et al.-Can J Cardiol |
| 103 | M | 3 | c.966_978del | exon 8 | frameshift | / | / | N | 3.0 | / | 2025-Zhang Q, et al.-Can J Cardiol |
| 104 | M | 20 | c.973_974insC | exon 8 | frameshift | / | / | Y | 7.7 | / | 2025-Zhang Q, et al.-Can J Cardiol |
| 105 | F | 10 | c.974del | exon 8 | frameshift | / | / | N | 2.5 | / | 2025-Zhang Q, et al.-Can J Cardiol |
| 106 | M | 16 | c.1003C>T | exon 8 | nonsense | / | / | N | 10.2 | / | 2025-Zhang Q, et al.-Can J Cardiol |
| 107 | M | 11 | c.1003C>T | exon 8 | nonsense | / | / | Y | 2.3 | / | 2025-Zhang Q, et al.-Can J Cardiol |
| 108 | M | 5 | c.1082del | exon 8 | frameshift | / | / | N | 3.1 | / | 2025-Zhang Q, et al.-Can J Cardiol |
| 109 | M | 1 | Xq24microdeletions | exon 1-9 | / | / | / | N | 7.6 | / | 2025-Zhang Q, et al.-Can J Cardiol |
| 110 | M | 1 | Xq24microdeletions | exon 1-9 | / | / | / | N | 7.6 | / | 2025-Zhang Q, et al.-Can J Cardiol |
| 111 | F | 14 | c.865-1G>C | intron 6 | splicing | / | N | / | / | / | 2025-Lu J, et al.-Cardiol Young |
| 112 | F | 7 | exon 4-9 | exon 4-9 | Loss of heterozygosity | / | N | / | / | / | 2025-Lu J, et al.-Cardiol Young |
| 113 | M | 12 | c.741+2T＞C | exon 6 | splicing | / | N | Y | 1.0 | N | 2025-Wang M, et al.-Chin J Hypertens |
| 114 | M | 15 | IVS6 + 1G > T | intron 6 | splicing | / | N | Y | / | / | 2026-Yu K, et al.-Medicine |
| 115 | M | 12 | exon 1 | exon 1 | deletion | / | N | / | / | / | 2026-Yu K, et al.-Medicine |

VP: ventricular pre-excitation
